# Bridging the gap in mental health knowledge and gender-based violence interventions: findings from a mixed-methods study in Moldova

**DOI:** 10.1192/j.eurpsy.2025.363

**Published:** 2025-08-26

**Authors:** V. Condrat, J. Chihai

**Affiliations:** 1 Trimbos Moldova; 2 Mental Health, Moldova State University of Medicine and Pharmacy, Chisinau, Moldova, Republic of

## Abstract

**Introduction:**

This study examines the intersection of mental health services and gender-based violence (GBV) in Moldova, identifying knowledge gaps and assessing service capacity through a mixed-methods approach, including focus groups and a national KAP survey. The findings highlight the urgent need for specialized training and evidence-based interventions to improve mental health outcomes and GBV responses.

**Objectives:**

The study aimed to develop targeted interventions and training programs to enhance the capacity of mental health professionals in Moldova to address mental health challenges and gender-based violence (GBV) by identifying knowledge gaps, assessing practices, and providing actionable insights for improved service delivery and outcomes.

**Methods:**

The study used a mixed-methods design, combining quantitative surveys and qualitative focus groups to assess knowledge gaps and training needs among mental health professionals in Moldova. A cross-sectional survey gathered quantitative data on perceptions of knowledge gaps, confidence, and training needs from a representative sample of professionals, including CCSM coordinators. Focus group discussions, held on the same day, provided qualitative insights into their challenges and suggestions for service improvement. This approach offered both breadth and depth, leading to evidence-based recommendations for addressing the identified gaps in knowledge and training.

**Results:**

The study uncovered major knowledge gaps among mental health professionals in Moldova, particularly in psychopharmacology, child and geriatric psychiatry, and gender-based violence (GBV) management. These gaps, identified via surveys and focus groups, reflected a lack of confidence in applying modern protocols and navigating legal aspects. There was a strong call for continuous professional development, especially through structured training in underserved regions. Inadequate training negatively affected the quality of care, particularly in GBV cases, though professionals were eager to improve their skills despite regional disparities in resources. The findings underscore the need for targeted training and better resource allocation.

**Conclusions:**

The study concluded that mental health professionals in Moldova face significant knowledge gaps, particularly in psychopharmacology, child and geriatric psychiatry, and managing gender-based violence (GBV). These gaps, along with insufficient training, negatively impact care quality, especially for GBV cases. Despite motivation to improve, professionals face challenges like limited access to updated training and regional disparities in resources, highlighting the need for structured, continuous training and equitable resource distribution to improve mental health services nationwide.

**Disclosure of Interest:**

None Declared

